# Modern Approaches to the Treatment of Acute Facial Pain

**DOI:** 10.1007/s11916-024-01260-4

**Published:** 2024-05-07

**Authors:** Auste Asadauskas, Markus M. Luedi, Richard D. Urman, Lukas Andereggen

**Affiliations:** 1 Department of Neurosurgery, Cantonal Hospital of Aarau, Aarau, Switzerland; 2 Faculty of Medicine, University of Bern, Bern, Switzerland; 3 Department of Anaesthesiology, Rescue- and Pain Medicine, Cantonal Hospital of St. Gallen, St. Gallen, Switzerland; 4 Department of Anaesthesiology and Pain Medicine, Inselspital, Bern University Hospital, University of Bern, Bern, Switzerland; 5 Department of Anesthesiology, The Ohio State University, Columbus, OH 43210 USA

**Keywords:** Acute pain; facial, Medical treatment, Intervention, Surgery

## Abstract

**Purpose of Review:**

Acute facial pain presents a complex challenge in medical practice, requiring a comprehensive and interdisciplinary approach to its management. This narrative review explores the contemporary landscape of treating acute facial pain, delving into pharmacological, non-pharmacological, and advanced interventions. The significance of tailored treatment strategies, rooted in the diverse etiologies of facial pain, such as dental infections, trigeminal neuralgia, temporomandibular joint disorders, sinusitis, or neurological conditions like migraines or cluster headaches, is underscored. We particularly emphasize recent advances in treating trigeminal neuralgia, elucidating current treatment concepts in managing this particular acute facial pain.

**Recent Findings:**

Recent research sheds light on various treatment modalities for acute facial pain. Pharmacotherapy ranges from traditional NSAIDs and analgesics to anticonvulsants and antidepressants. Non-pharmacological interventions, including physical therapy and psychological approaches, play pivotal roles. Advanced interventions, such as nerve blocks and surgical procedures, are considered in cases of treatment resistance. Moreover, we explore innovative technologies like neuromodulation techniques and personalized medicine, offering promising avenues for optimizing treatment outcomes in acute facial pain management.

**Summary:**

Modern management of acute facial pain requires a nuanced and patient-centric approach. Tailoring treatment strategies to the individual's underlying condition is paramount. While pharmacotherapy remains a cornerstone, the integration of non-pharmacological interventions is essential for comprehensive care. Advanced interventions should be reserved for cases where conservative measures prove inadequate. Furthermore, leveraging innovative technologies and personalized medicine holds promise for enhancing treatment efficacy. Ultimately, a holistic approach that considers the diverse needs of patients is crucial for effectively addressing acute facial pain.

## Introduction

**Fig. 1:**
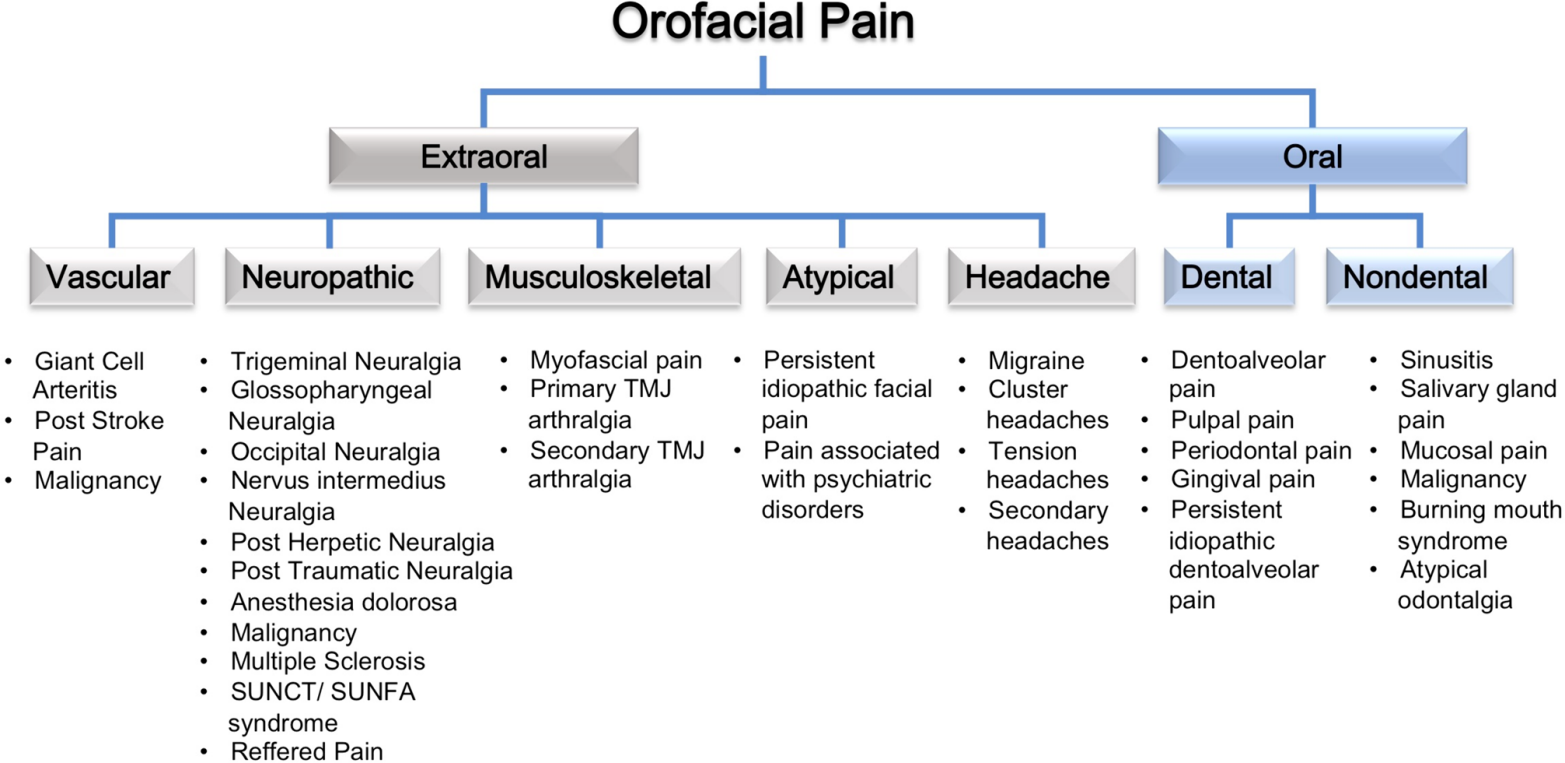
The differential diagnosis of facial pain encompasses various possibilities. Extraoral facial pain may arise from neuropathic disorders, vascular disorders, temporomandibular disorders, or atypical causes. Pain originating from within the mouth can be attributed to dental or nondental causes. Abbr.: TMJ, temporomandibular joint

The underlying causes of acute facial pain are diverse, encompassing conditions such as dental infections, trigeminal neuralgia (TN), temporomandibular joint disorders, sinusitis, migraines, and cluster headaches (Fig. 1) [1•, 2•, 3–5].

As such, effective management of acute facial pain in contemporary medicine necessitates a holistic approach that draws upon various specialized disciplines. Collaborative efforts spanning neurology, otolaryngology, neurosurgery, dentistry, and anesthesiological pain management are integral to address this complex condition comprehensively [2•, 5, 6]. Consequently, treatment protocols must be meticulously customized to address the specific underlying etiology of the pain [1•, 2•, 7–9]. The cornerstone of this approach lies in the utilization of both pharmacological and non-pharmacological interventions. Pharmacotherapy comprises a range of options, including nonsteroidal anti-inflammatory drugs (NSAIDs), analgesics, muscle relaxants, anticonvulsants, or antidepressants, tailored to the specific diagnosis and characteristics of the pain [10, 11]. Simultaneously, non-pharmacological interventions encompass physical therapy, acupuncture, chiropractic manipulation, and psychological approaches like cognitive-behavioral therapy (CBT), which can be initiated in the acute phase to identify and address associated anxiety or depression promptly [12–15]. When conservative measures prove insufficient in providing relief, advanced interventions merit consideration. These encompass nerve blocks, botulinum toxin injections, radiofrequency ablation, with microvascular decompression being particularly notable for conditions like trigeminal neuralgia (TN) [16, 17]. In recent years, the latter has gained prominence as a first-line treatment due to its high success rates and durable outcomes, even when neurovascular conflict is absent, provided that other underlying causes, such as tumors or multiple sclerosis lesions in the brainstem nucleus of the trigeminal nerve contributing to secondary TN, have been ruled out [18, 19••, 20••]. Additionally, advancements in medical technology have led to the development of innovative approaches for managing acute facial pain. Neuromodulation techniques such as transcutaneous electrical nerve stimulation (TENS) or peripheral nerve stimulation (PNS) can offer targeted pain relief with minimal side effects, although they are not used as primary treatment for acute pain symptoms [15, 17, 21]. Furthermore, the emergence of personalized medicine and precision diagnostics holds promise for optimizing treatment outcomes in patients with acute facial pain. By utilizing genetic testing, imaging modalities, and other biomarkers, clinicians can better tailor treatment approaches to individual patients, improving efficacy and minimizing adverse effects [6].

In this narrative review, we describe the intricate landscape of managing acute facial pain, stressing the importance of a comprehensive, interdisciplinary approach that encompasses various causes. In particular, we delve into modern treatment modalities, encompassing pharmacological, non-pharmacological, and advanced interventions, with particular attention to recent developments in addressing TN as a significant component in the array of acute facial pain disorders.

## Pharmacologic Treatments for Trigeminal Neuralgia

Treatment for TN includes pharmaceutical interventions, surgical procedures, and complementary methods [15, 20••], with anti-epileptic drugs typically utilized as the first-line treatment, [5, 16, 17, 22, 23] while secondary TN should be treated for underlying pathologies [5, 23]. Primary pharmacological options in initial therapy include sodium-blocking agents such as carbamazepine and oxcarbazepine, while lamotrigine, gabapentin, and baclofen are among the secondary options [16, 17, 23]. Sodium-blocking agents, notably carbamazepine, typically demonstrate superior efficacy in addressing paroxysmal firing. Conversely, gabapentinoids and antidepressants have shown effectiveness in managing persistent pain [17]. These alternative treatments can be employed independently or alongside other therapies as supplements [24, 81].

### Carbamazepine and Oxcarbazepine

Carbamazepine and oxcarbazepine, the latter being a structural keto derivative of the former, find extensive application in addressing a wide range of neuropathic pain conditions [16, 25]. Both medications extensively diminish the occurrence and severity of painful episodes, exhibiting equal efficacy in alleviating both spontaneous and trigger-induced attacks. Consequently, they provide significant initial pain relief to nearly 90% of patients [25, 26••, 27]. However, both drugs have a serious side effect profile, including hyponatremia, drug-drug interactions, aplastic anemia, and liver failure [20••]. Nevertheless, due to the high success in initial pain control, they remain the most effective medications, especially in the early stages of TN [28, 29]. In a comprehensive investigation involving typical instances of TN displaying resistance to carbamazepine treatment, it was observed that monotherapy with oxcarbazepine yielded additional pain alleviation in 37.1% of cases [30]. This finding suggests that oxcarbazepine holds promise as a viable recourse for patients encountering ineffectiveness with carbamazepine therapy, serving as a potential salvage strategy for those who do not derive relief from the standard treatment [20••]. It is crucial to note that while many patients initially respond to first-line therapy, most treatment approaches tend to diminish in efficacy over time, highlighting the need for new and innovative treatment options [31].

### Lamotrigine and Baclofen and Gabapentin

For TN patients who are intolerant of, or have contraindications to first line therapies, baclofen, lamotrigine or gabapentin are frequently viable alternatives [17, 20••, 28]. Moreover, patients who do not respond to first-line carbamazepine monotherapy have demonstrated potential benefits from combination therapy [20••, 32]. Nevertheless, there is a lack of randomized controlled trials directly comparing the efficacy of monotherapy against combination therapy in treating TN [28]. Each of these second line therapies come with certain pitfalls. Baclofen, a GABA_B_ receptor agonist, reduces the number of painful episodes and prolongs remission [20••]. The adverse effects of baclofen within a therapeutic window of up to 80 mg/day encompass drowsiness, muscle weakness, fatigue, and cognitive deficits. Additionally, the narrow therapeutic range of baclofen necessitates vigilant monitoring during dose initiation, with tapering recommended [20••, 33]. The effectiveness of baclofen is constrained by these adverse effects, often hindering the administration of an adequate oral dose required for meaningful pain alleviation [29]. Lamotrigine is an anticonvulsant that inhibits glutamate release by blocking voltage-gated sodium channels [20••, 24]. Within a therapeutic dosage range of up to 600 mg/day, its adverse effects may include sleepiness, dizziness, headache, vertigo, and ataxia [20••, 28, 34]. However, evidence supporting its efficacy is generally low [10, 34]. Gabapentin, an anticonvulsant designed to mimic the neurotransmitter GABA, lacks clear evidence regarding its effectiveness as monotherapy [20••]. A recent comprehensive examination involving a systematic review and meta-analysis, which scrutinized 18 randomized controlled trials, confirmed the efficacy and enhanced tolerability of this treatment in contrast to carbamazepine. However, the overall quality of the studies included in the analysis was deemed insufficient [35]. Due to the scarcity of evidence, it is understandable that some experts recommend early surgical referrals for patients unresponsive to first-line therapy, as they are less likely to respond to alternative medications for trigeminal neuralgia [28, 29].

### Alternative Pharmacotherapy

Several other drugs have demonstrated some evidence of efficacy for TN as well as other causes of acute facial pain in small, generally lower-quality controlled trials [28]. These include medications like pregabalin, pimozide, topical lidocaine, clonazepam, misoprostol, valproate, and others [11, 20••, 34]. However, due to a lack of longitudinal data, the long-term efficacy of these drugs still remains unknown.

### Analgesics

Many current treatment strategies prioritize long-term symptom management but often overlook the immediate relief of acute facial pain episodes. Rapid pain alleviation is essential for sudden and severe exacerbations of facial pain. Current recommendations advocate for the use of local anesthetics, particularly lidocaine (applied ophthalmically, intranasally, orally, via trigger point injection, administered intravenously, or utilized for nerve blocks), anticonvulsants (such as phenytoin or its derivative, fosphenytoin), and serotonin agonists (administered subcutaneously or intranasally, such as sumatriptan) [10, 36, 37].

## Botulinum Toxin Injections

Botulinum neurotoxin stands as a potent biological toxin and a formidable therapeutic agent, finding broadening utility across an expanding spectrum of clinical applications within the orofacial domain [38]. Its efficacy is particularly pronounced in addressing movement disorders, regulating saliva secretion, and managing both acute and neuropathic pain [39, 40]. Botulinum toxin exerts its pain-relieving effects through various mechanisms, including the reduction of inflammation, deactivation of sodium channels, and inhibition of axonal transport junctions [20••]. It has emerged as a novel and promising alternative to surgery for individuals whose pain is unresponsive to medication [41]. A recent systematic review and meta-analysis highlighted four small placebo-controlled randomized controlled trials assessing the use of botulinum toxin A for TN. The overall effect demonstrated an 85% reduction in TN attacks [42]. Botulinum toxin injections hold potential benefits for patients with TN and other orofacial disorders, although data remain limited at present.

## Interventional Treatments for Trigeminal Neuralgia

While medication currently stands as the primary approach for managing acute facial pain, [22, 23] prolonged pharmaceutical pain management presents drawbacks, as long-term usage is associated with various side effects [43]. Consequently, surgical intervention becomes a consideration when drug therapy proves ineffective or leads to intolerable side effects [22, 44, 45]. The European Academy of Neurology guidelines recommend medical management with appropriate doses and regular monitoring before considering surgery [17, 46•]. However, the optimal number of drugs before surgical referral has not yet been determined [47].

### Microvascular Decompression

Microvascular decompression (MVD) surgery stands as a well-established and effective treatment option for TN, intermedius neuralgia, hemifacial spasm, and glossopharyngeal neuralgia [18, 19••, 48].After Peter J. Jannetta reported promising results for MVD surgery in 1996, [49] it has become the surgical treatment of choice for TN. MVD is considered the primary surgical option and the only treatment addressing the etiology of classical TN [50, 51]. This surgical intervention tackles the challenge of vascular compression, which constitutes 85% of TN cases [19••].

**Fig. 2:**
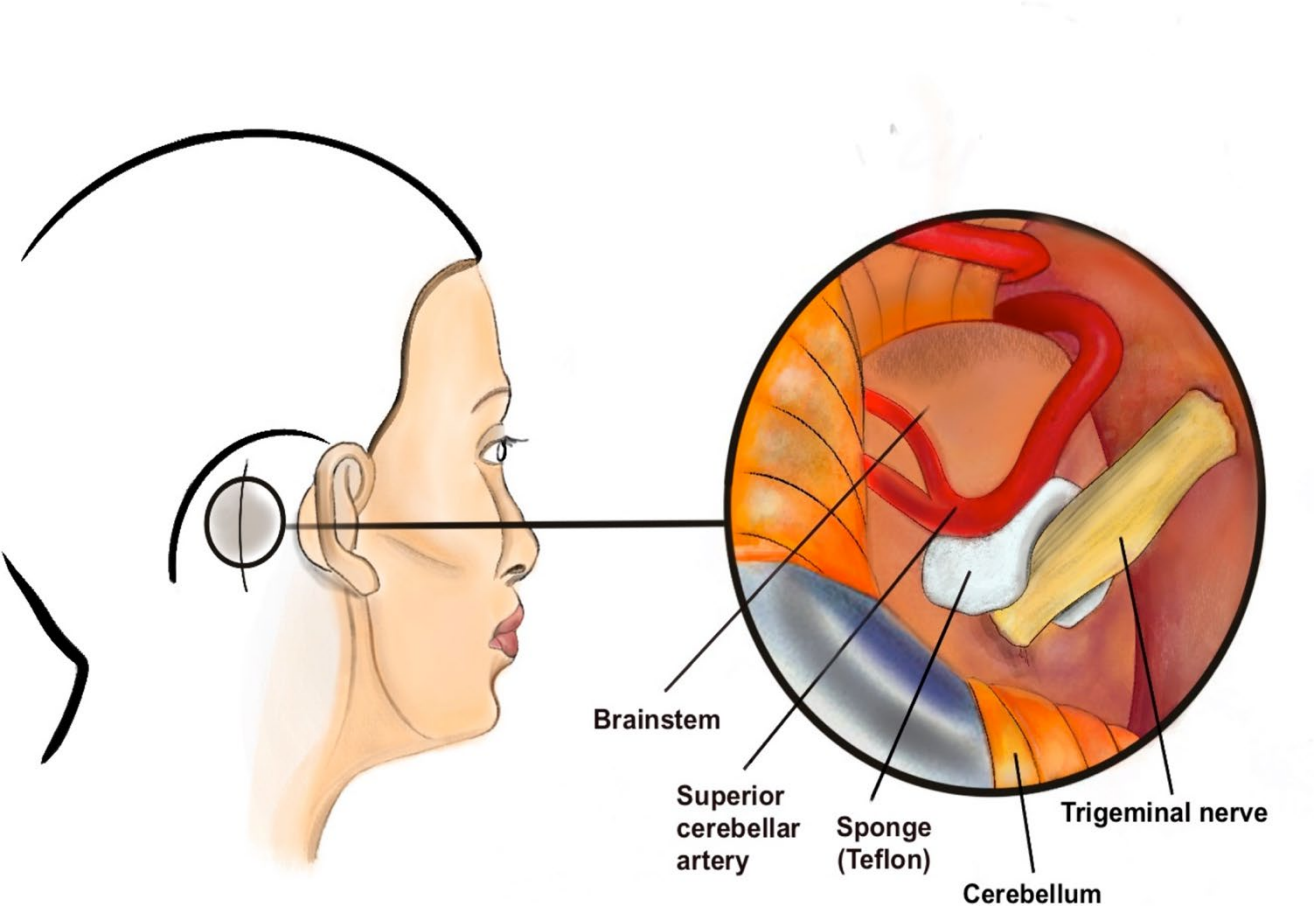
Microvascular decompression for TN. Retromastoid craniotomy is performed through a small incision behind the ear. Small pads of Teflon are placed between the trigeminal nerve and the artery (i.e. superior cerebellar artery) to decompress the nerve and preserve its normal function

For MVD, the retrosigmoid approach with a 3–4 cm surgical incision behind the ear and a 1.5–2 cm bone window is typically considered (Fig. 2) [50]. After opening the dura mater, cerebrospinal fluid is released, allowing for gentle retraction of the cerebellum to enhance visibility at the surgical site. Following this, the responsible blood vessels, typically the superior cerebellar artery, and the trigeminal nerve at its root entry zone are separated using Teflon (see Fig. 2) or, less commonly, a sling technique [50, 52–54]. Alternatively, transposition for MVD is an elegant way of solving vessel-nerve conflicts at the cerebellopontine angle, although a significant difference to interposition could not be proven [55].

Outcomes following MVD are generally positive, with systematic reviews indicating success rates exceeding 90% for the initial surgical treatment [51, 56–58]. Research suggests that patients who undergo MVD experience favorable long-term pain control outcomes, with approximately 70% of individuals becoming pain-free and no longer requiring medication a decade post-surgery [20••]. Moreover, those who undergo early MVD treatment express greater satisfaction compared to those opting for drug therapy [50, 59]. The recurrence rate ranges from 14 to 16% [44, 50, 59]. Although complications such as facial numbness, dull sensation, and vertigo are not uncommon, more severe complications like cerebral infarction, facial paralysis, hearing impairment, and infection are rare, occurring in less than 3% of cases [43, 56]. Full endoscopic vascular decompression has been suggested as a substitute for traditional MVD, providing similar therapeutic benefits with minimized surgical trauma, enhanced visibility of the surgical field, and potentially lower incidences of recurrence and complications [60]. However, the findings regarding its efficacy are varied.

### Percutaneous Balloon Compression

In recent year, percutaneous balloon compression (PBC) has emerged as a valuable therapeutic option in the management of TN [61–63]. This minimally invasive procedure is indicated for patients who have failed to respond to conservative treatments or are not suitable candidates for more invasive surgical interventions [20••, 62, 64]. Despite its poential association with nerve damage, it has been demonstrated that the therapeutic efficacy of PBC may be comparable to that of MVD, [62, 65] yet the long-term results are less favorable [66]. The surgery involves inserting a small catheter through the patient's cheek to access the Gasserian ganglion, either under fluoroscopic guidance or neuronavigation. Once the catheter is properly positioned, a small balloon is inflated to compress the trigeminal nerve, aiming to disrupt the abnormal pain signals responsible for TN [62, 64]. Studies have reported varying success rates with PBC, ranging from 80 to 90% in achieving pain relief and time free from medications from 2 to 3 years [63]. Recent studies demonstrate pain recurrence between 15 and 50% over 2–5 years [20••, 67]. Various factors have been identified as having an impact on the results of balloon compression. These factors encompass the shape of the inflated balloon, the pressure at which the balloon opens, the quantity of contrast injected, the duration of compression, and the specific underlying medical condition being treated [68, 69]. Of note, the procedure may result in facial sensations, cranial nerve deficits, and sudden heart rate changes due to trigeminal cardiac reflexes, although such occurrences are rare [20••, 69]. Nevertheless, compared to MVD, PBC offers shorter operation times, lower anesthesia risks, and the possibility of repeated procedures [61]. Nonetheless, it is noteworthy that there exists a notable incidence of trigeminal motor function impairment (66%), frequently prompting the need for repeated interventions owing to the heightened recurrence propensity [50, 64].

### Percutaneous Glycerol Infiltration

Percutaneous glycerol infiltration (PGC) or glycerol rhizotomy, is an alternative minimally invasive procedure used in the management of TN [19••, 20••]. Like for PBC, the primary indication is for patients who have not achieved sufficient relief from pharmacological treatments or who are not suitable candidates for MVD surgery [20••, 64]. Thereby, glycerol acts by damaging the trigeminal nerve fibers interrupting the abnormal pain signals responsible for TN [64]. Recent results indicate that glycerol rhizotomy displays a high short-term success rate, with over 90% of patients obtaining initial relief and over 50% of patients remaining pain free at three years [22]. However, MVD typically provides better and longer-lasting pain relief compared to percutaneous surgery, despite an increased risk of complications [70]. Balloon compression among percutaneous procedures offers the most durable pain relief [70]. Factors such as older age and post-operative numbness predict favorable outcomes from percutaneous surgery [70]. Currently, these findings can aid clinicians in the decision making to guide patients with primary TN regarding neurosurgical treatment choices for acute pain management.

### Radiofrequency Ablation

Although primarily employed for neuropathic facial pain, radiofrequency ablation (RF) utilizes thermocoagulation to selectively deactivate pain fibers [71, 72]. Conversely, RF has also been indicated and primarily utilized for urgent management when MVD is unsuitable for the patient [72]. Thereby, various RF techniques, such as conventional RF (CRF) and pulsed RF (PRF), differ in their mechanisms and efficacy in treating acute facial pain [17]. CRF produces an electric field of 5 to 15 mm, elevating tissue temperature above 45 °C, leading to localized damage and loss of nerve fibers, while PRF administers 20 ms pulses every 0.5 s, allowing for heat dissipation, thus avoiding temperatures exceeding 45 °C [17, 73]. Consequently, PRF inflicts less harm on surrounding tissues. For optimal outcomes and safety during RF ablation at the Gasserian ganglion, the triangular plexus, extending from the posterior margin of the ganglion to the path above the upper petrous ridge, proves to be the most advantageous site for generating precise lesions [17, 74]. Nevertheless, expected complications from RF ablation include pain recurrence, diminished corneal sensation, masseter weakness, dysesthesia, and other neurological issues [72]. A very recent review analyzing 1146 patients across 13 trials have demonstrated a success rate of 89.2% with RF ablation for TN [75]. While RF is superior to glycerol rhizotomy for immediate pain relief, it poses a higher risk of pain recurrence compared to MVD [76]. Despite its effectiveness in providing complete pain relief, particularly for high-risk surgical patients, evidence regarding the safety of RF ablation remains insufficient [17]. Yet, further research is warranted to conclusively establish the safety profile.

### Stereotactic Radiosurgery and Emerging Non-invasive Therapies

Although stereotactic radiosurgery (SRS) is not recommended for the immediate management of acute pain in TN, it can be employed as a supplementary therapy subsequent to the aforementioned procedures to prolong the efficacy, particularly in patients at elevated risk of recurrence or those considered unsuitable candidates for high-risk surgery [18, 77]. This procedure, conducted on an outpatient basis, entails administering high doses of radiation, reaching up to 80 Gy, through precise targeting of submillimeter radiation beams at the trigeminal root entry zone located in the posterior fossa [25, 78]. Over time, the targeted radiation induces necrosis, consequently diminishing pain signals [51]. Thereby, the most frequent complications reported are paraesthesias and facial numbness [51]. The literature is limited in its level of evidence, with only one comparative randomized trial [79]. A systematic review has illustrated a success rate of 69% at 1 year and 52% at 3 years post-surgery [22, 24, 51]. Overall outcomes were superior following MVD than following SRS, additionally a significantly higher amount stay without medication after MVD than after SRS [45]. Therefore, SRS is rarely utilized in the management of acute facial pain. In cases where the pain leans towards neuropathic pain, the utilization of deep brain stimulation has been documented to provide effective pain relief in 75–100% of patients undergoing treatment for neuropathic pain syndrome [22, 80]. However, two randomized controlled trials (RCTs) have failed due to study heterogeneity, suggesting that other less invasive methods are more promising [21, 22, 80].

Neuromodulation methods like transcutaneous electrical nerve stimulation (TENS) or peripheral nerve stimulation (PNS) present opportunities for precise pain relief with minimal adverse effects. However, they are typically employed as adjunctive rather than primary treatments for acute pain symptoms [15, 17, 21]. Moreover, the advancement of personalized medicine and precision diagnostics offers potential for enhancing treatment outcomes in individuals experiencing acute facial pain. Through the utilization of genetic testing, imaging techniques, and various biomarkers, healthcare professionals can customize treatment strategies to suit each patient's unique needs, thereby enhancing effectiveness and reducing unwanted effects [6].

In summary, the management of acute facial pain requires a comprehensive interdisciplinary approach, drawing upon various specialized disciplines. Treatment protocols must be meticulously customized to address the specific underlying etiology of the pain, utilizing both pharmacological and non-pharmacological interventions.

While pharmacological treatments such as carbamazepine and oxcarbazepine are available for trigeminal neuralgia (TN), advanced interventions like percutaneous balloon compression (PBC) and radiofrequency (RF) procedures present alternative options with differing effectiveness and safety characteristics, underscoring the need for additional research to establish definitive evidence [69]. Furthermore, MVD surgery represents a well-established and effective treatment option for TN, boasting high success rates and durable outcomes. However, it is essential to consider that multimorbid patients may not derive the same benefits from this option due to the potential perioperative complication rate. Neuromodulation techniques like TENS or PNS provide targeted pain relief, although not as primary treatments for acute pain, while personalized medicine and precision diagnostics are hypothesized to show promise in further optimizing treatment outcomes for acute facial pain through tailored interventions using genetic testing, imaging, and biomarkers.

## Conclusion

Modern approaches in treating acute facial pain encompass a comprehensive and interdisciplinary approach that integrates pharmacological, non-pharmacological, and advanced interventions. By addressing the underlying cause of the pain and utilizing a personalized treatment strategy, clinicians can effectively alleviate symptoms and improve the quality of life for patients experiencing acute facial pain.

## Data Availability

Not applicable.

## References

[1] • Zakrzewska JM. Differential diagnosis of facial pain and guidelines for management. Br J Anaesth. 2013;111(1):95–104. **A structured overview of differential diagnosis and management of facial pain.** 23794651 10.1093/bja/aet125

[2] • Romero-Reyes M, Uyanik JM. Orofacial pain management: current perspectives. J Pain Res. 2014;7:99–115. **A structured overview of differential diagnosis and management of orofacial demonstrating both non-pharmacological and pharmacological modalities.** 10.2147/JPR.S37593 PMC3937250 24591846

[3] Siccoli MM, Bassetti CL, Sándor PS. Facial pain: clinical differential diagnosis. Lancet Neurol. 2006;5(3):257–67. 16488381 10.1016/S1474-4422(06)70375-1

[4] Hegarty AM, Zakrzewska JM. Differential diagnosis for orofacial pain, including sinusitis, tmd, trigeminal neuralgia. Dent Update. 2011;38(6):396–408. 21905353 10.12968/denu.2011.38.6.396

[5] Yao AL, Barad M. Diagnosis and management of chronic facial pain. BJA Educ. 2020;20(4):120–5. 33456940 10.1016/j.bjae.2020.01.001 PMC7807944

[6] May A, Benoliel R, Imamura Y, et al. Orofacial pain for clinicians: A review of constant and attack-like facial pain syndromes. Cephalalgia 2023;43(8):3331024231187160. 10.1177/03331024231187160 37548299

[7] Bello C, Andereggen L, Luedi MM, Beilstein CM. Postcraniotomy headache: etiologies and treatments. Curr Pain Headache Rep. 2022;26(5):357–64. 35230591 10.1007/s11916-022-01036-8 PMC9061675

[8] Wipplinger F, Holthof N, Andereggen L, Urman RD, Luedi MM, Bello C. Meditation as an adjunct to the management of acute pain. Curr Pain Headache Rep. 2023;27(8):209–16. 37285010 10.1007/s11916-023-01119-0 PMC10403447

[9] Baumann L, Bello C, Georg FM, Urman RD, Luedi MM, Andereggen L. Acute pain and development of opioid use disorder: patient risk factors. Curr Pain Headache Rep. 2023;27(9):437–44. 37392334 10.1007/s11916-023-01127-0 PMC10462493

[10] Moore D, Chong MS, Shetty A, Zakrzewska JM. A systematic review of rescue analgesic strategies in acute exacerbations of primary trigeminal neuralgia. Br J Anaesth. 2019;123(2):e385–96. 31208761 10.1016/j.bja.2019.05.026 PMC6676170

[11] Minervini G, Franco R, Crimi S, et al. Pharmacological therapy in the management of temporomandibular disorders and orofacial pain: a systematic review and meta-analysis. BMC Oral Health. 2024;24(1):78. 38218874 10.1186/s12903-023-03524-8 PMC10787959

[12] Priyank H, Shankar Prasad R, Shivakumar S, et al. Management protocols of chronic orofacial pain: a systematic review. Saudi Dent J. 2023;35(5):395–402. 37520608 10.1016/j.sdentj.2023.04.003 PMC10373074

[13] Gupta R. Non-pharmaceutical management of chronic pain. GSC Advanced Research and Reviews. 2023;16(2):158–65. 10.30574/gscarr.2023.16.2.0112

[14] Nguyen CT, Wang MB. Complementary and Integrative Treatments. Otolaryngol Clin North Am. 2013;46(3):367–82. 23764815 10.1016/j.otc.2013.01.002

[15] Antony A, Antony AB, Mazzola AJ, Dhaliwal GS, Hunter CW. Comprehensive Review Neurostimulation for the Treatment of Chronic Head and Facial Pain: A Literature Review. Pain Physician [Internet]. 2019;22:447–77. Available from: https://www.painphysicianjournal.com/31561646

[16] Guo M, Shen W, Zhou M, et al. Safety and efficacy of carbamazepine in the treatment of trigeminal neuralgia: A metanalysis in biomedicine. Math Biosci Eng. 2024;21(4):5335–59. 38872538 10.3934/mbe.2024235

[17] Lee JY, Lee GH, Yi SH, Sim WS, Kim BW, Park HJ. Non-Surgical Treatments of Trigeminal Neuralgia from the Perspective of a Pain Physician: A Narrative Review. Biomedicines. 2023;11(8):2315. 37626811 10.3390/biomedicines11082315 PMC10452234

[18] Khan M, Nishi SE, Hassan SN, Islam MdA, Gan SH. Trigeminal Neuralgia, Glossopharyngeal Neuralgia, and Myofascial Pain Dysfunction Syndrome: An Update. Pain Res Manag. 2017;2017:1–18. 10.1155/2017/7438326 PMC5554565 28827979

[19] •• Park CK, Park BJ. Surgical Treatment for Trigeminal Neuralgia. J Korean Neurosurg Soc. 2022;65(5):615–21. **Review showing the outcomes of various surgical approaches in the treatment of trigeminal neuralgia.** 35430788 10.3340/jkns.2021.0265 PMC9452382

[20] •• Xu R, Xie ME, Jackson CM. Trigeminal Neuralgia: Current Approaches and Emerging Interventions. J Pain Res. 2021;14:3437–63. **Review about the current medical and surgical approaches in trigeminal neuralgia.** 34764686 10.2147/JPR.S331036 PMC8572857

[21] Qassim H, Zhao Y, Ströbel A, et al. Deep Brain Stimulation for Chronic Facial Pain: An Individual Participant Data (IPD) Meta-Analysis. Brain Sci. 2023;13(3):492. 36979302 10.3390/brainsci13030492 PMC10046035

[22] Jones MR, Urits I, Ehrhardt KP, et al. A Comprehensive Review of Trigeminal Neuralgia. Curr Pain Headache Rep. 2019;23(10):74. 31388843 10.1007/s11916-019-0810-0

[23] Di Stefano G, Truini A, Cruccu G. Current and Innovative Pharmacological Options to Treat Typical and Atypical Trigeminal Neuralgia. Drugs. 2018;78(14):1433–42. 30178160 10.1007/s40265-018-0964-9 PMC6182468

[24] Al-Quliti KW. Update on neuropathic pain treatment for trigeminal neuralgia. Neurosciences. 2015;20(2):107–14. 25864062 10.17712/nsj.2015.2.20140501 PMC4727618

[25] Lambru G, Zakrzewska J, Matharu M. Trigeminal neuralgia: a practical guide. Pract Neurol. 2021;21(5):392–402. 34108244 10.1136/practneurol-2020-002782 PMC8461413

[26] •• Cruccu G, Di Stefano G, Truini A. Trigeminal Neuralgia. N Engl J Med. 2020;383(8):754–62. **Shows the new classification fo trigeminal neuralgia and demonstrates in extensive detail the pharmacological management of trigeminal neuralgia.** 32813951 10.1056/NEJMra1914484

[27] Ma RSY, Kayani K, Oshodi DW, et al. Voltage gated sodium channels as therapeutic targets for chronic pain. J Pain Res. 2019;12:2709–22. 31564962 10.2147/JPR.S207610 PMC6743634

[28] Xu R, Xie ME, Jackson CM. Trigeminal Neuralgia: Current Approaches and Emerging Interventions. J Pain Res. 2021;14:3437–63. 34764686 10.2147/JPR.S331036 PMC8572857

[29] Cruccu G. Trigeminal Neuralgia. CONTINUUM: Lifelong Learning in Neurology. 2017;23(2):396–420. 28375911 10.1212/CON.0000000000000451

[30] Gomez-Arguelles JM, Dorado R, Sepulveda JM, et al. Oxcarbazepine monotherapy in carbamazepine-unresponsive trigeminal neuralgia. J Clin Neurosci. 2008;15(5):516–9. 18378142 10.1016/j.jocn.2007.04.010

[31] Obermann M. Recent advances in understanding/managing trigeminal neuralgia. F1000Res. 2019;8:505. 10.12688/f1000research.16092.1 PMC6480942 31069052

[32] Zakrzewska JM, Linskey ME. Trigeminal neuralgia. BMJ. 2014;348(feb17 9):g474–g474. 24534115 10.1136/bmj.g474

[33] Romito JW, Turner ER, Rosener JA, et al. Baclofen therapeutics, toxicity, and withdrawal: A narrative review. SAGE Open Med. 2021;9:205031212110221. 10.1177/20503121211022197 PMC8182184 34158937

[34] Rana MH, Khan AAG, Khalid I, et al. Therapeutic Approach for Trigeminal Neuralgia: A Systematic Review. Biomedicines. 2023;11(10):2606. 37892981 10.3390/biomedicines11102606 PMC10604820

[35] De Stefano G, Di Pietro G, Truini A, Cruccu G, Di Stefano G. Considerations When Using Gabapentinoids to Treat Trigeminal Neuralgia: A Review. Neuropsychiatr Dis Treat. 2023;19:2007–12. 37745191 10.2147/NDT.S407543 PMC10517700

[36] Ananthan S, Quek SYP, Baddireddy SM, Zagury JG. Use of injection techniques in orofacial pain emergencies: a narrative review. J Oral Maxillofac Anesth. 2022;1:26–26. 10.21037/joma-22-11

[37] Peterson-Houle GM, AbdelFattah MR, Padilla M, Enciso R. Efficacy of medications in adult patients with trigeminal neuralgia compared to placebo intervention: a systematic review with meta-analyses. J Dent Anesth Pain Med. 2021;21(5):379. 34703889 10.17245/jdapm.2021.21.5.379 PMC8520835

[38] Ghurye S, McMillan R. Orofacial pain – an update on diagnosis and management. Br Dent J. 2017;223(9):639–47. 29074941 10.1038/sj.bdj.2017.879

[39] Serrera-Figallo M-A, Ruiz-de-León-Hernández G, Torres-Lagares D, et al. Use of Botulinum Toxin in Orofacial Clinical Practice. Toxins (Basel). 2020;12(2):112. 32053883 10.3390/toxins12020112 PMC7076767

[40] Val M, Delcanho R, Ferrari M, Guarda Nardini L, Manfredini D. Is Botulinum Toxin Effective in Treating Orofacial Neuropathic Pain Disorders? A Systematic Review. Toxins (Basel). 2023;15(9):541. 37755967 10.3390/toxins15090541 PMC10535201

[41] Kayani AMA, Silva MS, Jayasinghe M, et al. Therapeutic efficacy of botulinum toxin in trigeminal neuralgia. Cureus. 2022;14(7):e26856. 10.7759/cureus.26856 PMC9375637 35974855

[42] Anton R, Juodzbalys G. The use of botulinum toxin a in the management of trigeminal neuralgia: a systematic literature review. J Oral Maxillofac Res 2020;11(2):e2. 10.5037/jomr.2020.11202 PMC7393930 32760475

[43] Holste K, Chan AY, Rolston JD, Englot DJ. Pain Outcomes Following Microvascular Decompression for Drug-Resistant Trigeminal Neuralgia: A Systematic Review and Meta-Analysis. Neurosurgery. 2020;86(2):182–90. 30892607 10.1093/neuros/nyz075 PMC8253302

[44] Mizobuchi Y, Nagahiro S, Kondo A, et al. Microvascular Decompression for Trigeminal Neuralgia: A Prospective. Multicenter Study Neurosurgery. 2021;89(4):557–64. 34325470 10.1093/neuros/nyab229

[45] Inoue T, Hirai H, Shima A, et al. Long-term outcomes of microvascular decompression and Gamma Knife surgery for trigeminal neuralgia: a retrospective comparison study. Acta Neurochir (Wien). 2017;159(11):2127–35. 28905114 10.1007/s00701-017-3325-7

[46] • Bendtsen L, Zakrzewska JM, Abbott J, et al. European Academy of Neurology guideline on trigeminal neuralgia. Eur J Neurol. 2019;26(6):831–49. **Demonstrates the new guidelines regarding trigeminal neuralgia and the importance of a multidisciplinary approach.** 30860637 10.1111/ene.13950

[47] Bendtsen L, Zakrzewska JM, Heinskou TB, et al. Advances in diagnosis, classification, pathophysiology, and management of trigeminal neuralgia. Lancet Neurol. 2020;19(9):784–96. 32822636 10.1016/S1474-4422(20)30233-7

[48] Peng W, Zhao R, Guan F, et al. Fully endoscopic microvascular decompression for the treatment of hemifacial spasm, trigeminal neuralgia, and glossopharyngeal neuralgia: a retrospective study. BMC Surg. 2023;23(1):331. 37891595 10.1186/s12893-023-02214-0 PMC10612333

[49] Kaufmann AM, Price AV. A history of the Jannetta procedure. J Neurosurg. 2020;132(2):639–46. 10.3171/2018.10.JNS181983 30717044

[50] Yu G, Leng J, Xia Y, Min F, Xiang H. Microvascular decompression: Diversified of imaging uses, advantages of treating trigeminal neuralgia and improvement after the application of endoscopic technology. Front Neurol 2022;13:1018268. 10.3389/fneur.2022.1018268 PMC9681918 36438943

[51] Araya EI, Claudino RF, Piovesan EJ, Chichorro JG. Trigeminal Neuralgia: Basic and Clinical Aspects. Curr Neuropharmacol. 2020;18(2):109–19. 31608834 10.2174/1570159X17666191010094350 PMC7324879

[52] Sade B, Lee JH. Microvascular Decompression for Trigeminal Neuralgia. Neurosurg Clin N Am. 2014;25(4):743–9. 25240661 10.1016/j.nec.2014.06.007

[53] Hatipoglu Majernik G, Wolff Fernandes F, Al-Afif S, Heissler HE, Krauss JK. Microsurgical posterior fossa re-exploration for recurrent trigeminal neuralgia after previous microvascular decompression: common grounds-scarring, deformation, and the “piston effect.” Acta Neurochir (Wien). 2023;165(12):3877–85. 37955684 10.1007/s00701-023-05877-z PMC10739219

[54] Menna G, Rapisarda A, Izzo A, et al. Surgical and Clinical Outcomes of Microvascular Decompression: A Comparative Study between Young and Elderly Patients. Brain Sci. 2022;12(9):1216. 36138952 10.3390/brainsci12091216 PMC9496765

[55] Uhl C, Faraj L, Fekonja L, Vajkoczy P. Transposition versus interposition method in microvascular decompression for trigeminal neuralgia: midterm analysis of both techniques in a single-center study. J Neurosurg 2024;1–8. 10.3171/2023.11.JNS231658 38277665

[56] Pascasio LA, De La Casa-Fages B, De Antonio EE, Grandas F, García-Leal R, Juretschke FR. Microvascular decompression for trigeminal neuralgia: A retrospective analysis of long-term outcomes and prognostic factors. Neurología. 2023;38(9):625–34. 37996213 10.1016/j.nrl.2021.03.009

[57] Sharma R, Phalak M, Katiyar V, Borkar S, Kale S, Mahapatra A. Microvascular decompression versus stereotactic radiosurgery as primary treatment modality for trigeminal neuralgia: A systematic review and meta-analysis of prospective comparative trials. Neurol India. 2018;66(3):688. 29766927 10.4103/0028-3886.232342

[58] Liu J, Chen Z, Feng T, Jiang B, Yuan Y, Yu Y. Biomedical Glue Sling Technique in Microvascular Decompression for Trigeminal Neuralgia Caused by Atherosclerotic Vertebrobasilar Artery: A Description of Operative Technique and Clinical Outcomes. World Neurosurg. 2019;128:e74-80. 30954750 10.1016/j.wneu.2019.03.289

[59] Jiao L, Ye H, Lv J, et al. A Systematic Review of Repeat Microvascular Decompression for Recurrent or Persistent Trigeminal Neuralgia. World Neurosurg. 2022;158:226–33. 34875391 10.1016/j.wneu.2021.11.129

[60] Zagzoog N, Attar A, Takroni R, Alotaibi M, Reddy K. C.01 Endoscopic versus open microvascular decompression of trigeminal neuralgia: a systematic review and comparative meta-analysis. Can J Neurol Sci. 2018;45(s2):S13-4. 10.1017/cjn.2018.96 30544341

[61] Xu B, Jia Z, Ren H, et al. Clinical Efficacy of a Spiral CT-Guided Balloon Compression Day-Surgery Operation for the Treatment of Trigeminal Neuralgia. Front Neurol 2022;13:923225. 10.3389/fneur.2022.923225 PMC9298876 35873781

[62] Deng S, Luo J, Lai M, et al. Percutaneous balloon compression for trigeminal neuralgia: experience and surgical techniques from a single institution. Acta Neurol Belg. 2023;123(6):2295–302. 37353706 10.1007/s13760-023-02310-1 PMC10682111

[63] Valenzuela Cecchi B, Figueroa F, Contreras L, Bustos P, Maldonado F. Percutaneous Balloon Compression for the Treatment of Trigeminal Neuralgia: A Review of 10 Years of Clinical Experience. Cureus. 2023;15(8):e43645. 10.7759/cureus.43645 PMC10505044 37719619

[64] Chang KW, Jung HH, Chang JW. Percutaneous Procedures for Trigeminal Neuralgia. J Korean Neurosurg Soc. 2022;65(5):622–32. 35678088 10.3340/jkns.2022.0074 PMC9452389

[65] Ni H, Wang Y, Chen X, Gu W. Outcomes of Treatment for Elderly Patients With Trigeminal Neuralgia: Percutaneous Balloon Compression Versus Microvascular Decompression. J Craniofac Surg. 2020;31(7):e685–8. 32472880 10.1097/SCS.0000000000006544

[66] Wu J, Xiao Y, Chen B, Zhang R, Dai M, Zhang Y. Efficacy and safety of microvascular decompression versus percutaneous balloon compression in the treatment of trigeminal neuralgia: a systematic review and meta-analysis. Ann Palliat Med. 2022;11(4):1391–400. 35523747 10.21037/apm-21-3901

[67] Abdennebi B, Guenane L. Technical considerations and outcome assessment in retrogasserian balloon compression for treatment of trigeminal neuralgia. Series of 901 patients. Surg Neurol Int. 2014;5(1):118. 25101213 10.4103/2152-7806.137838 PMC4123256

[68] Noorani I, Lodge A, Vajramani G, Sparrow O. Comparing Percutaneous Treatments of Trigeminal Neuralgia: 19 Years of Experience in a Single Centre. Stereotact Funct Neurosurg. 2016;94(2):75–85. 27071078 10.1159/000445077

[69] Xia Y, Yu G, Min F, Xiang H, Huang J, Leng J. The Focus and New Progress of Percutaneous Balloon Compression for the Treatment of Trigeminal Neuralgia. J Pain Res. 2022;15:3059–68. 36199499 10.2147/JPR.S374433 PMC9529012

[70] Noorani I, Lodge A, Durnford A, Vajramani G, Sparrow O. Comparison of first-time microvascular decompression with percutaneous surgery for trigeminal neuralgia: long-term outcomes and prognostic factors. Acta Neurochir (Wien). 2021;163(6):1623–34. 33751217 10.1007/s00701-021-04793-4 PMC8116280

[71] Elawamy A, Abdalla EEM, Shehata GA. Effects of Pulsed Versus Conventional Versus Combined Radiofrequency for the Treatment of Trigeminal Neuralgia: A Prospective Study. Pain Physician. 2017;20(6):E873–81. 28934792

[72] Wang Z, Wang Z, Li K, Su X, Du C, Tian Y. Radiofrequency thermocoagulation for the treatment of trigeminal neuralgia. Exp Ther Med. 2021;23(1):17. 34815769 10.3892/etm.2021.10939 PMC8593925

[73] Nguyen M, Wilkes D. Pulsed Radiofrequency V2 Treatment and Intranasal Sphenopalatine Ganglion Block: A Combination Therapy for Atypical Trigeminal Neuralgia. Pain Pract. 2010;10(4):370–4. 20492576 10.1111/j.1533-2500.2010.00382.x

[74] Wu H, Zhou J, Chen J, Gu Y, Shi L, Ni H. Therapeutic efficacy and safety of radiofrequency ablation for the treatment of trigeminal neuralgia: a systematic review and meta-analysis. J Pain Res. 2019;12:423–41. 30697063 10.2147/JPR.S176960 PMC6342144

[75] Eskandar E, Kumar H, Boini A, et al. The role of radiofrequency ablation in the treatment of trigeminal neuralgia: a narrative review. Cureus 2023;15(3):e36193. 10.7759/cureus.36193 PMC10104592 37065382

[76] Yan C, Zhang Q, Liu C, et al. Efficacy and safety of radiofrequency in the treatment of trigeminal neuralgia: a systematic review and meta-analysis. Acta Neurol Belg. 2022;122(4):1019–30. 33988820 10.1007/s13760-021-01654-w

[77] Warnick RE, Paddick I, Mathieu D, et al. The relevance of biologically effective dose for pain relief and sensory dysfunction after Gamma Knife radiosurgery for trigeminal neuralgia: an 871-patient multicenter study. J Neurosurg. 2024;1–13. 10.3171/2023.12. 10.3171/2023.12.JNS231569 38364220

[78] Smith ZA, Gorgulho AA, Bezrukiy N, et al. Dedicated linear accelerator radiosurgery for trigeminal neuralgia: a single-center experience in 179 patients with varied dose prescriptions and treatment plans. Int J Radiat Oncol Biol Phys. 2011;81(1):225–31. 21236592 10.1016/j.ijrobp.2010.05.058

[79] Tuleasca C, Régis J, Sahgal A, et al. Stereotactic radiosurgery for trigeminal neuralgia: a systematic review. J Neurosurg. 2019;130(3):733–57. 10.3171/2017.9.JNS17545 29701555

[80] Buchanan RJ, Darrow D, Monsivais D, Nadasdy Z, Gjini K. Motor cortex stimulation for neuropathic pain syndromes. NeuroReport. 2014;25(9):715–7. 24780896 10.1097/WNR.0000000000000174

[81] Maniam R, Kaye AD, Vadivelu N, Urman RD. Facial Pain Update: Advances in Neurostimulation for the Treatment of Facial Pain. Curr Pain Headache Rep. 2016;20(4):24. 26896948 10.1007/s11916-016-0553-0

